# Knowing How to Ask About Digital Culture in Youth Mental Health Care: A Co‐Designed Tool

**DOI:** 10.1111/eip.70203

**Published:** 2026-06-24

**Authors:** Vincent Paquin, Rebecca Jiang, Jai L. Shah, G. Eric Jarvis, Elizabeth Nickrenz, Manuela Ferrari, Melissa Park

**Affiliations:** ^1^ Department of Psychiatry McGill University Montreal Québec Canada; ^2^ Lady Davis Institute for Medical Research Jewish General Hospital Montreal Québec Canada; ^3^ Douglas Mental Health University Institute Montreal Québec Canada; ^4^ Department of Psychology Duquesne University Pittsburgh Pennsylvania USA; ^5^ School of Physical & Occupational Therapy McGill University Montreal Québec Canada

## Abstract

**Background:**

With the digital cultures that youth are exposed to and participating in come potential risks and protective factors for their mental health. However, despite clear need there is a lack of guidance to help mental health professionals evaluate the role of social media, artificial intelligence, and other technologies in young people's mental health.

**Aims:**

To co‐design with young people the Digital Culture Interview, an interview tool to support the clinical assessment of digital cultural factors in mental health care.

**Method:**

We recruited a diverse group of 12 participants aged 16–35 years (mean age 22 years) from outpatient mental health clinics in Montreal, Canada. Using the nominal group technique, they identified topics they found most relevant for exploring in a clinical assessment the experiences and practices involving digital technologies. Based on the topics that received the most votes from participants, we co‐developed a list of interview questions and written guidance for their administration.

**Results:**

Participants identified and ranked 48 themes. Drawing from these, 14 questions were developed for inclusion in the Digital Culture Interview, covering four topics: identity and worldview, negative experiences online, coping, and understanding of mental health. Participants emphasised that exploring digital culture in mental health care requires patients' trust and a baseline of knowledge. If done sensitively, this may enhance the patient‐clinician alliance and improve mutual understanding.

**Conclusions:**

The Digital Culture Interview has the potential to enhance rapport and reveal risk and protective factors that are salient to and actionable in mental health care.

## 1 Introduction

In Canada, young people aged 16–25 years and ‘older youth’ aged 26–35 years spend the most time on digital media compared to other age groups (Government of Canada, S. C 2024; MHRC 2024, 19), and they are the first to uptake new technologies such as chatbots, TikTok, and virtual reality (Buckle 2019; Ipsos 2024; Made in CA 2024). Understanding the impacts of digital technologies at that age is important because most (75%) mental health problems emerge by age 35 (Solmi et al. 2022), and intervening on social and lifestyle factors can prevent or mitigate mental health problems (Salazar de Pablo et al. 2021). Indeed, the evolving landscape of artificial intelligence, social media, and video games raises widespread concerns about the impacts of technologies on youth mental health: for example, artificial intelligence chatbots like ChatGPT and Character.AI are thought to have contributed to instances of delusional thinking and suicidal behaviours (Chung et al. 2026); social media use is associated with negative social comparisons, inattention, impaired sleep, and loneliness (Capraro et al. 2025); and gaming disorder is recognised by the World Health Organization as a persistent pattern of video gaming that is hard to control and persists despite negative psychosocial consequences (WHO 2024). Thus, exposure to these and other digital media experiences constitutes potential risk factors for mental health problems among adolescents and young adults.

Like many technologies, however, digital technologies are not just associated with harms but also present several potential benefits. In some circumstances, interaction with artificial intelligence chatbots can mitigate loneliness (Fang et al. 2025) and provide meaningful support (Luo et al. 2025). Social media allows people to maintain relationships across distances and access safe spaces for self‐expression and communication (Popat and Tarrant 2023). Video games can have restorative effects on mood and help players fulfil their needs for autonomy, competence, and social connectedness (Ballou et al. 2025). The distinct features of online communication, such as searchability, asynchronicity, and anonymity, enable alternative means for people to express their difficulties, find like‐minded peers, and obtain information about diagnoses and treatments (Rains 2020). Importantly, the uses and impacts of digital technologies vary as a function of individual and social contexts. People from LGBTQ+ communities (Berger et al. 2022) and people with disabilities (Rains 2018), for example, may particularly benefit from online communication for finding safe spaces for self‐expression and peer support. On the other hand, being from a minority or marginalised group puts one at greater risk of harassment and discrimination in online spaces (Imperato and Mancini 2025).

Despite widespread concerns for digital‐related risks, mental health professionals feel ill‐equipped to address them. In a sample of 113 psychiatry residents in the United Kingdom, 50.4% observed digital‐related mental health issues in their patients, but the majority (67.3%) did not feel competent to assess these issues (Aref‐Adib et al. 2020). This sentiment is shared by psychiatrists, family physicians, and other youth workers (Derges et al. 2023; Million et al. 2025; Rifkin‐Zybutz et al. 2023). Although there are clinical tools and guidelines that can help clinicians assess screen time limits (Tremblay et al. 2016) and diagnose gaming disorder (WHO 2024), these resources only capture a small part of digital media experiences. Other guides for clinical interviews propose broader assessments (Carson et al. 2018; Moreno et al. 2024); however, they were developed based on expert opinion without formally consulting young people, making it unclear to what extent they reflect the experiences and priorities of youth. Indeed, when youth are asked by clinicians about their use of digital technologies, most (58%) feel judged or misunderstood (Rifkin‐Zybutz et al. 2023). A sensitive approach to this assessment, reflecting the experiences and priorities of young people, holds the potential to improve the patient‐clinician alliance and help intervene on digital‐related risk and protective factors (Jarvis et al. 2020; Paquin et al. in press).

To facilitate this, the current project aimed to develop an interview tool for the clinical assessment of digital media experiences in mental health care. We co‐designed the tool with people aged 16–35 years and followed in outpatient mental health clinics to reflect their priorities and experiences. This article reports on the co‐design process and its outcomes: key topics and guidance for the assessment of digital media experiences in mental health care, and the integration of these insights into a novel interview tool—the Digital Culture Interview (DCI).

### 1.1 Theoretical Framework

This project was guided by a cultural psychiatry framework, which recognises the influence of sociocultural factors related to identity, illness understanding (e.g., how mental distress is explained), sources of stress and resilience (e.g., bullying and community support), and the effect of the patient‐clinician relationship (e.g., fear of stigma) on clinical presentation and treatment (Aggarwal and Lewis‐Fernández 2015; Jarvis and Kirmayer 2021; Kleinman 1981). This lens encourages clinicians and researchers to understand people's experiences on their own terms, and as such we used a participatory co‐design approach to develop the Digital Culture Interview (Hagen et al. 2012). To capture how the varied impacts of digital technologies go beyond individual behaviours, we employ the concept of digital culture. Digital culture refers not only to platforms, but also to the shared practices, social norms, and expectations—such as the pressure to be available online, conform to the latest trends, and maintain a successful online image—that shape how people engage with digital media (Thumim 2012). Digital culture also includes subcultural elements, such as intimate relationships with artificial intelligence agents, online self‐diagnosis promoted by algorithms, conspiracy beliefs, and influencers that shape identity, social ties, and sources of stress and resilience (Paquin 2025). The concept is different from digital literacy, which describes competence in using and critically thinking about digital media (Buckingham 2015), whereas digital culture centers on meaning‐making and lived experience.

## 2 Methods

### 2.1 Study Design

This co‐design initiative, which took place between April and September 2025, involved a combination of group and individual discussions. The full protocol and materials have been published elsewhere (Paquin et al. 2025). Reflecting the aim of the DCI and the context in which its use is intended, we sought co‐design participants with lived experience of mental health problems and of undergoing clinical assessments. To be eligible, participants had to be 16–35 years old and followed in outpatient mental health clinics in Montreal, Canada. Participants were also required to speak English and French, reflecting common bilingualism (58.5% of the population) in Montreal (Government of Canada 2022). We chose to form a bilingual group to avoid privileging one language over the other and to mirror the cultural composition of the city, with the caveat that populations who are less likely to be bilingual (e.g., recent immigrants) may have been underrepresented. Other eligibility criteria were clinical stability according to the treating team, reported usage of digital technologies (social media, online videos, or video games) at least multiple times a week, and lack of intellectual disability. The study received ethical approval from the Research Ethics Board of the CIUSSS de l'Ouest‐de‐l'Île‐de‐Montréal (MP‐18‐2025‐1164), and all participants provided written informed consent.

Participants were recruited using a purposive sampling strategy, aiming for diversity across genders and diagnostic categories (including but not restricted to psychotic, affective, neurodevelopmental, and personality disorders). We contacted mental health professionals and joined their team meetings to solicit their help in presenting the study to patients who may be interested. We additionally displayed flyers inviting interested individuals to contact us directly. All participants were met with individually to present the study objectives and review the consent form. Our goal was to recruit approximately 10 participants—a number large enough to provide a diversity of perspectives, but small enough to allow group discussions. We approached 21 people, of whom 5 were not interested and 4 could not be reached, leading to a final group of 12 (Table 1).

**TABLE 1:** Descriptive information about co‐design participants (*N* = 12).

Descriptor	Mean (SD) or *N* (%)
Age, years	21.9 (5.0)
Gender	
Man	5 (41.7%)
Nonbinary	1 (8.3%)
Woman	6 (50.0%)
Self‐reported diagnosis or focus of mental health care^a^	
Anxiety	3 (25%)
Attention deficit and hyperactivity disorder	2 (16.7%)
Autism	2 (16.7%)
Bipolar disorder	1 (8.3%)
Depression	5 (41.7%)
Psychotherapy	3 (25%)
Psychotic disorder	2 (16.7%)
Ethnoracial groups	
Asian	2 (16.7%)
Black	3 (25.0%)
Mixed	2 (16.7%)^b^
White	5 (41.7%)
Employed and/or student	
Yes	7 (58.3%)
No	5 (41.7%)

^a^ Some participants gave multiple answers.

^b^ ‘Hispanic and mixed’ and ‘Latinx and White’.

### 2.2 Positionality

We recognise that researchers' backgrounds and social positions influence research, including interactions with participants, power imbalances, and the interpretation of data. The first author and lead researcher on the project, VP, is a psychiatrist in his 30s, White settler, born to a middle‐class French‐Canadian family. His research, guided by a critical realist paradigm (Paquin 2025), focuses on digital culture and youth mental health. The second author, RJ, co‐moderated the co‐design meetings. She is a medical student in her 20s, born to a middle‐class immigrant Chinese family, whose interest in digital culture and mental health research is guided by her experience as a health and lifestyle content creator. Other team members, representing various backgrounds and disciplines—cultural psychiatry, digital mental health, youth mental health, psychology, ethnography, and occupational therapy, as well as various forms of engagement with digital cultures—contributed to the development of the protocol and the interpretation of findings.

### 2.3 Co‐Design Process

The co‐design process was structured around two group meetings. The first meeting employed the nominal group technique to generate ideas for the content of the DCI. This technique consists in inviting participants to write down their ideas silently, then present their ideas one at a time in turn, until all ideas are presented (Harvey and Holmes 2012). At the end, participants vote on the ideas they find most important or relevant for the project. The advantage of the nominal group technique, relative to a focus group, is that it ensures equal opportunities for participation. To guide the generation of ideas, we instructed participants to think about the beneficial and harmful effects of digital media practices, as well as the impact that digital media can have on identity, understandings of mental illness and well‐being, and the patient‐clinician relationship.

Based on the ideas deemed most relevant by participants, our research team produced a first draft of the DCI. We shared it with participants by email, then revised it with participants during the second group meeting. Participants were invited to remove, revise, or add elements, and to provide feedback on the co‐design process. Throughout the co‐design process, participants unable to attend the group meetings were invited to individual interviews.

### 2.4 Analysis

We conducted a rapid thematic analysis (Braun and Clarke 2012) after the initial nominal group discussion to synthesise participants' answers into a list of (micro‐)themes that participants could subsequently rank by importance. This analysis was conducted by VP and reviewed with RJ using word processor software. VP began by listening to recordings of the nominal group discussions and revising the automatic transcriptions generated by Microsoft Teams. He inductively coded all responses from participants, attributing one or multiple codes per response, then extracted all codes and generated themes. VP and RJ revised the themes together and organised them by categories for presentation to participants. A second thematic analysis was conducted by VP and RJ at the end of the co‐design process to inductively identify themes related to two pre‐specified categories: participants' perspective on how the DCI should be administered, and the anticipated impacts of using the DCI in clinical practice.

## 3 Results

### 3.1 Generation of Themes

The first group meeting was 1.5 h. Of 12 participants, two were unable to attend and were met with individually, including one in‐person meeting at the clinic (all other interviews were virtual). A third participant could not attend but provided feedback by email, leaving nine participants in the group. We had initially planned to collect participants' votes during this first group meeting; however, due to time constraints and because some participants were absent, we opted for soliciting participants' votes by email after the meeting. This also allowed us to conduct a rapid thematic analysis to more thoroughly synthesise their answers.

A total of 48 themes were identified, which we organised into eight categories: online communities (eight themes), distraction and coping (four themes), online safety (six themes), algorithms and misinformation (five themes), problematic use (eight themes), social pressures (three themes), mental health information (eight themes), and the patient‐clinician relationship (six themes). The full list of themes is in Table S1.

This list was sent to participants by email, and participants were instructed to rank, in order, the five themes they found most relevant to explore in an initial clinical encounter. We converted their rankings into scores, so that a first rank weighs five points, a second rank weighs four points, and so on, which we then summed across participants to identify the highest‐ranked themes. As a starting point, we selected the top 15 themes, which all had a score of five points or more. We chose this number because we were aiming for an interview tool that takes about 30 min to administer and estimated that 15 themes would amount to about 15 questions or 30 min. Other themes not included in the top 15 were later reviewed with participants to consider additional elements for inclusion in the DCI.

### 3.2 Drafting the Interview

As a first draft of the DCI, VP prepared questions covering the 15 themes and organised them into categories. Given some overlap between the 15 themes, this first draft included 11 questions. Participants were sent by email the draft accompanied by the 15 themes that the questions were meant to capture (Table S2). We subsequently held the second group meeting, which lasted 1 h, to solicit their revisions to the draft, including to adjudicate on the transition from themes to DCI items, adjust the wording and structure of the questions, and incorporate additional content as needed based on the initial 48 themes or new insights. Participants could see the draft and list of themes during the meeting through screen sharing, and VP's note‐taking on the draft was visible to all, allowing for member checking. Participants were sent the revised draft again after the meeting to solicit further feedback. Seven attended the group meeting, two were unable to attend and were met with individually instead, one left the study, and two others could not be reached.

Participants made several suggestions, leading to extensive changes to the wording of all questions, elaboration on some of the questions, the merging of two questions, and the addition of four questions. Participants emphasised the importance of distinguishing between digital content that the patient may be *intentionally* seeking out on the internet from content that is *presented* to them by algorithms. They suggested asking not only about upsetting content seen online, but also inspiring content. In a question about being targeted or bullied online, they noted that the word “bullied” can carry a strong emotional connotation, and they advised asking instead about ‘people online who are treating you poorly’ as a more inclusive phrasing that might be less likely to cause distress. Participants recommended the addition of a question about the pressure to conform to online trends and norms. They also noted that it would be helpful to contrast how a person expresses their identity and copes with challenges online compared to offline. To better explore the impacts of scrolling, which could include negative emotional experiences as well as fatigue and attentional impairments, participants suggested inquiring about effects that are felt ‘physically, emotionally, or mentally’. After those interviews with participants, we invited clinicians and researchers on the team to provide their feedback, leading to final revisions to make the questions more open‐ended and to reorder them for flow. The resulting interview tool includes 14 questions (Table 2). Exemplars based on the themes generated by participants are included alongside the interview questions to provide further guidance. The DCI is a living toolkit, and eventual revisions will be made available via the Open Science Framework: https://osf.io/3g7zc/.

**TABLE 2:** The Digital Culture Interview.

Guide for the clinician	Questions for the patient
Introduction	
This interview should be adapted as needed.	Digital technologies sometimes influence people's lives and what they are going through, in good or bad ways.
Keep an open mind: some digital practices may seem unusual but are not necessarily harmful.	By digital technologies, I mean things like social media, apps, video games, online videos, and artificial intelligence.
To elicit more details, ask for examples.	I will ask you some questions about your experiences with digital technologies.
As the patient‐clinician alliance develops over time, the patient might share more. Therefore, it can be helpful to revisit these questions ulteriorly.	There is no right or wrong answer, and you can tell me as much or as little as you like.
Opening	
The exemplars below on the left (in italics) were provided by youth and are not intended to be exhaustive.	1. What is the role of digital technologies in your life?
Identity and worldview	
*The person might use digital technologies to explore and define their identity*.	2. How do you express your identity online? How does that compare to how you express it in person?
*Online contents often reflect a person's hobbies and interests, but some contents expose people to deception, false information, and echo chambers*. *Online news and algorithms may affect how the person sees the world*.	3. What kinds of content do you typically seek out on the internet? 4. What kinds of content get shown to you by social media? 5. What things do you see or hear about online that upset you? That inspire you?
Negative experiences online	
*The person might be concerned that they are addicted to digital technologies or that their use is getting in the way of in‐person relationships*.	6. Do you ever feel like you're struggling to control your use of digital technologies?
*The person might report “doomscrolling”: consuming large quantities of content on social media, often negative news. They might also be affected by violent or explicit content online*.	7. How does scrolling affect you, physically, emotionally, or mentally?
*The person might be targeted, harassed or bullied online, or might worry this could happen to them*.	8. Are there people online who are treating you poorly?
*The person might feel bad about themself because of impossible standards that they see online*.	9. Are there standards, trends, or norms online that you feel like you have to follow or should follow?
Coping	
*The person might use digital technologies for entertainment or comforting contents that help them feel better*.	10. In what ways do you use digital technologies to feel better or to deal with what you are going through?
*Digital technologies might provide various experiences, including distraction and escape*.	11. How do these experiences compare to what you can get outside of the digital world?
*Online social connections might help the person deal with mental health problems*. *The person might also find comfort in knowing others going through similar difficulties*.	12. Are there people or communities online that are providing you support or helping you deal with what you are going through?
Understanding of mental health	
*The person might use digital technologies to better understand their mental health and what they are going through*. *However, they might worry that the clinician will react defensively to information found on the internet*. *There is also a risk of inaccurate information online*.	13. Have you found information online that has helped you understand what you are going through? For example, regarding possible mental health diagnoses or treatments.
Conclusion	
Sharing digital contents might help the person communicate things that are meaningful to them. Based on the individual's response, the clinician can invite the person to show the content directly, if they want to. The clinician can also share related contents (e.g., memes) in response.	14. Is there a piece of digital content that you feel represents you or what you are going through? For example, it could be memes, content creators that you like, music, art, or conservations that you've had with AI.

### 3.3 Guidance for Administering the Interview

Beyond the questions that compose the DCI, participants voiced suggestions and concerns regarding how the questions should and should not be used. Their guidance, detailed below, was embedded directly in the interview toolkit, alongside the interview questions and exemplars.

Participants highlighted that youth digital culture is a complex topic that requires a sensitive exploration. The DCI should be administered flexibly by clinicians, adapting its length and using follow‐up questions as needed to further explore patients' experiences. Some questions may need to be revisited beyond the initial interview to allow trust to develop between the patient and the clinician. The person may anticipate being judged or facing negative repercussions for disclosing their digital media experiences and practices:

Let's say the patient is into some deep conspiracy theory or whatever. They can be afraid, and they might be not that willing to share what they think or what they know. (Participant 2)

Reflecting on marginalised or unusual contents that young people may seek online, many participants insisted on the importance of a non‐judgemental, open‐minded exploration:

Each person is different. Their preferences are all different, subjective to them. And I guess as long as it's not harmful, like no matter what they believe, what they see, what they like, it's like their right to do. [The clinician should] understand differences of people as long as they're not doing anything wrong. (Participant 11)

It was underscored that clinicians should not jump to conclusions about the person's unusual interests or habits involving digital technologies. Clinicians must be wary of being influenced by societal stigma surrounding some digital practices, such as young people's use of artificial intelligence chatbots as social companions and for psychological support.

Participants noted that clinicians often lack knowledge about young people's online spaces and practices. Consequently, clinicians may not have a good understanding of what patients are disclosing in response to the DCI's questions. In turn, a lack of understanding of patients' digital preferences and habits may lead to difficulties in differentiating problematic and non‐problematic behaviours, with the risk of harmless activities being pathologised by clinicians.

A patient's openness about their digital media practices will depend on their relationship with the clinician, including how long they have been working together and whether the clinician is deemed trustworthy. One of the participants suggested that, to foster a better understanding of patients' experiences, clinicians should familiarise themselves with online spaces and acquire a baseline of knowledge. In addition, clinicians can build trust and reciprocate these disclosures by sharing digital content that is relevant to what the person is discussing: for example, if the person mentions a *meme* (an internet‐based humoristic image), the clinician can show another one in return. Importantly, clinicians should recognise when the patient is not comfortable discussing a particular topic and allow them not to answer. At the same time, clinicians should not forgo delicate questions: asking directly about topics such as online self‐diagnosis, where patients may assume clinicians' disapproval, is necessary to open a space for discussion.

### 3.4 Anticipated Impacts of the Digital Culture Interview

Participants stressed the potential impact that improving clinicians' awareness of digital culture might have on the patient‐clinician alliance:

If clinicians just have like a general understanding of what a forum is or what a meme is and what some of these terms mean, to then have that to be built upon in a patient‐clinician relationship, it feels like that would also create this same feeling that I have here in discussing online spaces, where there's a fundamental baseline that we all kind of have. (Participant 7)

They observed that some questions in particular, such as those about digital contents sought online and online self‐diagnosis, might open conversations about topics that are meaningful to the patients yet often unexplored in clinical care. One participant noted the questions' broad relevance for understanding young people's experiences with digital technologies but was uncertain as to how the clinical exploration of these topics might influence clinical management. Overall, participants perceived the DCI as potentially impactful, but also potentially intrusive, requiring a careful use by the clinician.

## 4 Discussion

Through a combination of group discussions, individual interviews, and email exchanges, we developed with young people a clinical interview tool addressing the role of digital cultural factors in mental health. To our knowledge, this is the first interview tool designed with youth to explore the impacts of digital technologies on mental health in clinical care, beyond a single technology (e.g., social media) or metric (e.g., screen time). Drawing on a sociocultural perspective, its content reflects the experiences and knowledge of a diverse group of young people receiving mental health services. The hope is that by using the DCI, clinicians may better understand the digital lived experiences of youth, enhance their alliance with patients and better identify a person's unique risk and protective factors for mental health.

Participants emphasised that patients may worry about being stigmatised or misunderstood for their digital practices and preferences, echoing the experiences of young people reported elsewhere (Plackett et al. 2026; Rifkin‐Zybutz et al. 2023). Indeed, internet‐related practices are often framed negatively in popular discourses, particularly when it comes to youth culture (Orben 2020), and the stigma is even greater for internet subcultural communities that diverge from the mainstream (Paquin et al. 2026). To support a non‐judgemental exploration of the patient's experiences with digital technologies, the DCI includes questions not only about negative experiences, but also positive ones, such as coping and online support, and provides explicit instructions to the clinician (e.g., ‘Keep an open mind: some digital practices may seem unusual but are not necessarily harmful’; Table 2). However, this content does not fully address young people's fear of being misunderstood. To manage sensitive disclosures, clinicians may consider employing normalizing statements (e.g., ‘Some people see AI as a tool, while others develop a deep attachment to their chatbot; how is it like for you?’), open‐ended inquiries about motivations and positive experiences (e.g., ‘What do you like about interacting with this application?’), and validation (e.g., ‘I can understand how this interaction has helped you go through a difficult period’) (Shea 2016). More broadly, we believe that the implementation and use of the DCI should be accompanied by training to better address preconceptions among clinicians that are potentially stigmatizing or pathologizing.

The DCI's holistic exploration of positive and negative experiences is consistent with the view that an assessment of digital technology use should not be exclusively focused on problematic use or screen time, and that it should also be open to exploring digital experiences that are beneficial for a person's well‐being (Hamati et al. 2025; Looi et al. 2024; Moreno et al. 2024). Reflecting important threads of the literature on digital media effects, the DCI addresses the role of digital culture in identity development (Granic et al. 2020), negative social comparisons online (Gill 2023; van der Wal et al. 2024), harmful contents promoted by algorithms (Brady et al. 2023; Hamilton et al. 2025), and the addictive use of digital technologies (Brand et al. 2025). It explores interpersonal factors, including the use of digital technologies for creating and maintaining supportive social connections, which can be especially important for coping with illness and marginalisation (Berger et al. 2022; Rains 2018). Interpersonal stressors, such as the exposure to discrimination and harassment online (Nickerson and Fredrick 2025), as well as the displacement of in‐person interactions in favour of screen time (Capraro et al. 2025), are also captured by the interview questions and exemplars. Finally, the DCI's open‐ended questions may support the exploration of newer, rapidly evolving phenomena, such as the use of artificial intelligence companions for coping with mental health problems and loneliness (Laestadius et al. 2024; Luo et al. 2025), as well as shifting representations of diagnoses and treatments on social media (Alper et al. 2025).

The DCI rests on a concept of digital culture that ties together social media, video games, artificial intelligence, and other technologies and media forms. Although generally distinct, these technologies participate in a broader media ecology (Scolari 2012) and sometimes operate through shared pathways: online relationships can be formed in video games and exported to social media; smartphones, streaming, gaming, and social media can be implicated in forms of problematic (addictive) use that interferes with functioning (Brand et al. 2025); and a person's affinity for chatbots or influencers may draw them to online communities who share their interest (Paquin et al. 2026). Other affordances are more technology‐specific, such as social comparisons on social media (Orben et al. 2024), artificial sociality with chatbots (Depounti and Natale 2025), and interactive rewards in video games (Flayelle et al. 2023). Clinicians should consider both common and specific mechanisms for identifying the mental health risks and benefits of digital practices and their implications for intervention.

Overall, these themes represent factors that can act as predisposing, precipitating, perpetuating, or protective factors in a clinical presentation. By supporting their personalised evaluation in clinical care, the DCI may orient intervention approaches that mitigate harmful exposures while promoting and reinforcing the patient's resources. For example, a person's deep involvement in video games could indicate an affinity for tabletop roleplay games, which can be used as psychotherapeutic devices (Billieux et al. 2025; Henrich and Worthington 2023). Someone's difficulty with controlling their use of social media may contribute to depressive symptoms and interfere with plans for behavioural activation, prompting a tailored intervention to limit usage (Plackett et al. 2023). Another person's close relationship with a social chatbot could be a source of concern yet reveal an effective strategy for improving social skills (Franze et al. 2023). And as participants highlighted, if clinicians do not ask, patients may not tell.

A key limitation is that, owing to the geographical focus, the recruitment strategy and the small size of the co‐design group, the experiences and priorities identified in this project cannot reflect those of all youth. Populations in low‐income countries, recent immigrants, and youth with intellectual disability, for example, were not represented and may have varying practices and views related to digital technologies. A person's access to and engagement with digital culture is shaped by their languages (including whether they speak local majority languages), the prescription of specific digital practices within cultural communities (e.g., using or abstaining from using technologies for religion; Campbell and Tsuria 2021), and patterns of digital help‐seeking and literacy related to one's psychosocial needs and (dis)abilities. Accordingly, following the participants' call for a personalised administration of the DCI, some questions may be more or less relevant depending on a patient's unique context, and we encourage an intersectional approach to digital culture that accounts for the person's identities and sociocultural background. Another consideration is the influence of our positionality on the co‐design process. A potential strength is that the discussions were moderated by two people close in age to the study participants, with hands‐on knowledge of online spaces popular among youth. This background provided some degree of insider's perspective and may have facilitated rapport, though it also required the researchers to bracket their assumptions when listening to and interpreting participants' answers. In addition, given the first author's position as researcher and physician, participants might have prioritised discourses on technologies that they deemed scientifically and socially acceptable, to the detriment of more controversial views. Marginalised topics such as pornography use and the Dark Web, for instance, were not discussed. However, participants did criticise mainstream clinical practices, and they emphasised the risk of stigma towards youth digital practices. The next step of this project will be to test the DCI with an independent sample of people followed in outpatient mental health clinics and train clinicians in evaluating the digital lived experiences of their patients. We will collect feedback from patients and clinicians to explore the implications of the DCI for youth mental health care and examine its potential for implementation in clinical practice (Paquin et al. 2025).

## 5 Conclusion

This article presented the development of the DCI, an interview tool co‐designed with young people to support the assessment of digital media experiences and practices in clinical care. The interview guide covers a range of positive and negative experiences related to digital technologies and emphasises the importance of a non‐judgemental, flexible approach to clinical assessment. Potential impacts, to be examined in future research, are that the DCI can enhance rapport and support personalised interventions in youth mental health care.

## Funding

The authors have nothing to report.

## Conflicts of Interest

The authors declare no conflicts of interest.

## Supporting information


**Table S1:** List of themes generated from the first co‐design meeting and sent to participants for their votes.
**Table S2:** Highest‐ranked themes generated by participants and initial interview items.

## Data Availability

The data that support the findings of this study are available on request from the corresponding author. The data are not publicly available due to privacy or ethical restrictions.
