# Supplementary material for: Knowing How to Ask About Digital Culture in Youth Mental Health Care: A Co‐Designed Tool
Source: Early Interv Psychiatry. 2026 Jun 24;20(7):e70203. doi: 10.1111/eip.70203 (PMC13291760; doi:10.1111/eip.70203)
Supplement: Supplementary file 1 — Table S1: List of themes generated from the first co‐design meeting and sent to participants for their votes. Table S2: Highest‐ranked themes generated by participants and initial interview items. [file EIP-20-0-s001.docx]

**Supplement Table 1: List of themes generated from the first co-design meeting and sent to participants for their votes**

*Instruction to participants:*

Let’s say a professional is providing mental health care for an adolescent or young adult. What aspects of the patient’s experience with digital media could be the most relevant to discuss?

From the list below, please indicate the 5 statements you think could be the most relevant. Mark your votes as 1, 2, 3, 4, 5 in the column on the right, with 1 being the most relevant of all. You can distribute your votes wherever, as long as you use each number (1 to 5) only once.

| Statement | Vote |
| --- | --- |
| **Online communities** | |
| The person might use digital media to explore and define their identity. |  |
| The person might use digital media to learn interesting things from others. |  |
| Online social connections and communities might help the person deal with mental health problems. |  |
| There might be positive influences or people online that the person looks up to. |  |
| The person might feel a sense of community though digital media. |  |
| The person might receive support online for their mental health difficulties. |  |
| The person might find comfort in knowing others online who are going through similar mental health difficulties. |  |
| On social media, the person’s mental health problems might be treated as not real or not valid. |  |

| **Distraction and coping** | |
| --- | --- |
| The person might use digital media as an escape or distraction. |  |
| The person might use digital media for entertainment or comforting contents that help them feel better. |  |
| The person might use specific apps or AI to cope with their difficulties. |  |
| The person might have a personal relationship with AI such as ChatGPT. |  |

| **Online safety** | |
| --- | --- |
| The person might be exposed to (or be a source of) hateful speech online. |  |
| The person might be targeted, harassed or bullied online. |  |
| The person might be affected by violent or explicit content that they see online. |  |
| The person might be exposed to sexual exploitation online. |  |
| The person might learn habits or behaviors online that negatively impact their well-being. |  |
| The person might be affected by their personal information being leaked or sold online. |  |

| **Algorithms and misinformation** | |
| --- | --- |
| The person’s identity and worldview might be influenced by algorithms. |  |
| The person might be exposed to deception and false information online. |  |
| The person might develop extreme political views because of digital media. |  |
| The person might have beliefs that are hard to change because of digital media and echo chambers. |  |
| The person can protect themself by learning how algorithms work. |  |

| **Problematic use** | |
| --- | --- |
| The person might be addicted to digital media, such as social media, apps, and video games. |  |
| Doomscrolling might have a negative effect on the person’s mental health. |  |
| Because of the immediate satisfaction provided by digital media, the person might feel emotionally numb. |  |
| Constant interaction with digital media might affect the person’s memory and attention. |  |
| The person might be spending a lot of time on the internet to cope with loneliness. |  |
| The person’s use of digital media might get in the way of real-life relationships. |  |
| The person might have less real-life interaction with friends or families because of their use of digital media. |  |
| The person can learn to control digital media, rather than being controlled by it. |  |

| **Social pressures** | |
| --- | --- |
| The person might feel bad about themself because of impossible standards that they see online. |  |
| The person might feel pressure to follow trends because of digital media. |  |
| Pressure to fit in a community online might be a source of stress for the person. |  |

| **Mental health information** | |
| --- | --- |
| The person might use digital media to better understand their mental health and what they are going through. |  |
| The person might find explanations or diagnoses online for their mental health difficulties. |  |
| Algorithms might present the person with a lot of content related to their mental health problems. |  |
| The person’s mental health problem might be reinforced after seeing content about it online. |  |
| Digital media might help the person’s community have better awareness of mental health problems and reduce stigma. |  |
| Digital media might make the person’s community see mental health conditions as not serious. |  |
| The person might get inaccurate information about mental health or diagnoses online. |  |
| The person might have difficulty finding reliable sources of information online. |  |

| **Patient-clinician relationship** | |
| --- | --- |
| The person might rely on social media, apps, or video games to deal with health problems instead of seeing their treating team. |  |
| Because of information they obtained online, the person might disagree with their treating team about their diagnosis. |  |
| Health professionals might react defensively if the person brings up information that they found on the internet. |  |
| The person might have less trust in their treating team because of information they saw online. |  |
| If the person uses AI as a personal therapist, they might be less engaged with their treating team. |  |
| It might be difficult for health professionals to help change the person’s habits with digital media. |  |

**Supplement Table 2: Highest-ranked themes generated by participants and initial interview items**

| **Themes generated by participants** | **Interview items generated by the research team based on the themes** |
| --- | --- |
| *Worldview and identity* | |
| - The person might use digital media to explore and define their identity. | Are there aspects of your identity that you have been exploring or expressing online? |
| - The person might be exposed to deception and false information online. - The person might have beliefs that are hard to change because of digital media and echo chambers. - The person’s identity and worldview might be influenced by algorithms. - The person might be affected by violent or explicit content that they see online. | What kinds of contents do you typically look at on the internet?  Are there specific things that social media and algorithms tend to show you?  Are there things that you're seeing or hearing about online that are making you upset? For example, about politics or what is going on in the world. |
| *Negative experiences online* | |
| - Doomscrolling might have a negative effect on the person’s mental health. - The person’s use of digital media might get in the way of real-life relationships. - The person might feel bad about themself because of impossible standards that they see online. | Are there aspects of scrolling on social media that are causing you difficulties? |
| - The person might be targeted, harassed or bullied online. | Have you been targeted, harassed or bullied online? |
| *Coping* | |
| - The person might use digital media for entertainment or comforting contents that help them feel better. - The person might use digital media as an escape or distraction. | Are there apps, online videos, AI, or video games that you use to feel better or to deal with your difficulties? |
| - The person might find comfort in knowing others online who are going through similar mental health difficulties. - Online social connections and communities might help the person deal with mental health problems. | Do you know other people online who are going through similar difficulties? Is it helpful? Not helpful?  Are there people or communities online that are providing you support or helping you deal with your difficulties? |
| *Knowledge about mental health* | |
| - The person might use digital media to better understand their mental health and what they are going through. | Have you found information online that has helped you understand your difficulties? |
| *Control over digital media* | |
| - The person can learn to control digital media, rather than being controlled by it. | Sometimes people feel like digital technologies are controlling their lives. How is it for you, and what kinds of strategies do you have to stay in control of digital technologies? |

Some themes map onto multiple items and vice-versa, as represented by their grouping within the same rows.
